# What infection control measures will people carry out to reduce transmission of pandemic influenza? A focus group study

**DOI:** 10.1186/1471-2458-9-258

**Published:** 2009-07-23

**Authors:** Leanne G Morrison, Lucy Yardley

**Affiliations:** 1 School of Psychology, University of Southampton, University Road, Southampton, Hampshire, UK

## Abstract

**Background:**

Pandemic influenza poses a future health threat against which infection control behaviours may be an important defence. However, there is little qualitative research examining perceptions of infection control measures in the context of pandemic influenza.

**Methods:**

Eight focus groups and one interview were conducted with a purposive sample of 31 participants. Participants were invited to discuss their perceptions of infection transmission and likely adherence to infection control measures in both non-pandemic and pandemic contexts. Infection control measures discussed included handwashing, social distancing and cough hygiene (e.g. covering mouth, disposing of tissues immediately etc.).

**Results:**

Thematic analysis revealed that although participants were knowledgeable about infection transmission, most expressed unfavourable attitudes toward control behaviours in non-pandemic situations. However, with the provision of adequate education about control measures and appropriate practical support (e.g. memory aids, access to facilities), most individuals report that they are likely to adhere to infection control protocols in the event of a pandemic. Of the behaviours likely to influence infection transmission, handwashing was regarded by our participants as more feasible than cough and sneeze hygiene and more acceptable than social distancing.

**Conclusion:**

Handwashing could prove a useful target for health promotion, but interventions to promote infection control may need to address a number of factors identified within this study as potential barriers to carrying out infection control behaviours.

## Background

The risk of a pandemic outbreak of influenza is considered high. The World Health Organisation estimates that over seven million deaths worldwide may result [1]. Several behaviours have been recommended in order to limit the spread of influenza in the event of a pandemic [2]. These include handwashing [3-5], social distancing [6,7] and cough and sneeze hygiene measures.

A number of surveys have been carried out in order to examine factors associated with adherence to these behaviours. Particular attention has been paid to improving the handwashing practices of healthcare workers (HCWs) [8,9] and understanding implementation of infection control procedures during previous epidemics such as Severe Acute Respiratory Syndrome (SARS) [10-13]. This research has identified a positive association between implementation of infection control behaviour and knowledge, efficacy beliefs and risk perceptions [10,12,14-19]. Perceived personal risk appears to be an important factor. During the SARS epidemic, a survey of cross-border travellers from Hong Kong to mainland China identified that reported levels of mask wearing were lower when individuals stated they wore masks mainly in order to protect others from infection than when they wore masks to protect themselves [13]. However, the role of these factors may be less influential in the context of infections such as influenza, which is seen by most people as a familiar and everyday risk [20,21].

Research also points to socio-demographic factors as influences on infection control behaviour. Men were less likely than women to engage in appropriate handwashing practices in hospitals [14] and less likely to believe that preventive behaviours for controlling SARS were efficacious [11-13,18]. Findings with regard to age are more inconsistent, with some research identifying an inverted-U relationship [11] and some reporting that older adults are more likely to engage in certain types of preventive behaviours [12]. Practical barriers reported to impede implementation of infection control behaviour have included: high work load; time constraints; skin irritation; and availability of hand decontamination facilities [22,23].

While surveys are useful for identifying broad associations between preventive behaviour and attitudinal and sociodemographic factors, qualitative research is needed in order to develop a more detailed understanding of how these factors may affect adherence to infection control behaviours in different contexts. Qualitative research to date has focussed on the attitudes and perceptions of HCWs toward infection control behaviours [24,25] and continuing to work during a pandemic [26,27]. Whitby, McLaws, and Ross [25] found that nurses viewed handwashing as a habitual behaviour primarily determined by the perceived risk of infection from a patient i.e. primarily motivated by self-protection. The attitudes and motivations of HCWs may not be generalisable to individuals at a population level however, since they work within a health-specific context which actively promotes handwashing behaviour.

Three qualitative investigations were undertaken during the SARS epidemic examining individuals' experiences of quarantine [28,29] and the responses of older Chinese people living in Canada [30]. These studies suggest that social and emotional factors may override the influence of rational attitudes such as risk perceptions and efficacy beliefs. Older Chinese people held a perceived ethical duty to protect others, 'filial piety', which facilitated adherence to quarantine protocols [30]. However, an ethical duty could also act as a barrier to social isolation measures, with reports that caring for an infected loved one took priority over one's own health [28]. Research examining the handwashing behaviours of caregivers in developing countries also suggests that an immediate need to care for or "nurture" a child may undermine the implementation of adequate handwashing procedures [31]. Fears of isolation, boredom or stigmatisation were also barriers to compliance with infection control procedures [29].

We could only identify one qualitative study that has examined perceptions of pandemic influenza and infection control behaviours. Janssen, Tardif, Landry, and Warner [32] found that existing knowledge and awareness of pandemic influenza amongst the general public in the United States was on the whole very poor, with concern about a pandemic low. Few individuals were willing to learn and implement behaviours to control a pandemic when such an event was neither present nor showed immediate signs of occurring. This highlights the importance of context. Attitudes and responses at the height of a pandemic may vary considerably from responses when there is no salient threat of infection. Anticipated responses to the risk of a human-to-human H5N1 outbreak were also examined in Hong Kong, where people have recent experience with SARS [17]. The likelihood of adopting infection control behaviours was associated with older age, perceptions of risk and susceptibility to infection, perceived likelihood of a local outbreak, perceived severity as compared to SARS, perceived efficacy of the behaviours and concern for family members. However, it is unclear how far these findings can be generalised from non-western cultures such as Hong Kong and China to the UK and other western populations [11,17].

The research presented here examined perceptions of influenza and in particular the anticipated likelihood of implementing a variety of infection control behaviours in a Western culture with no recent epidemic experience. This study comprised the first empirical stage of the development of a complex intervention [33]. The purpose was to inform the development of a website-based infection control intervention to modify respiratory infection transmission within the home, in both non-pandemic and pandemic contexts. Following the PRECEDE-PROCEED intervention development approach [34], a medical team identified the behavioural determinants considered important to preventing virus transmission. The aim of this study was to determine the 'changeability' of these determinants, i.e. how acceptable and feasible it would be to promote each behaviour. A qualitative design was used so as to develop a detailed understanding of the interrelated factors which might support or inhibit the adoption of each of the infection control measures. Focus groups were carried out in order to facilitate natural interaction, comparison and discussion between participants.

## Methods

### Participants

Thirty one participants (18 women and 13 men) aged between 17 and 68 were recruited from Southern England. Criteria for recruitment were that participants were currently living with at least one other person and were fluent English speakers. Participants were recruited to the study using advertisements (paper-based and online) and snowballing techniques. Purposive sampling methods were used to ensure a diverse sample, including older people, people with and without children and people with varied living arrangements and relationship status (see Table 1 for a summary of participant characteristics).

**Table 1:** Summary of participant characteristics

Characteristic	*n *= 31
Gender: female *n *(%)	18 (58)
Age: M (SD), range	30.32 (14.34), 17–68
Relationship to other members of the household: *n *(%)	
Living with spouse/partner	12 (39)
Living with parent(s) and/or siblings	11 (35)
Living with friend(s) and/or unrelated people	7 (23)
Living with family relations	1 (3)
Share a bedroom: Yes *n *(%)	13 (42)
Possibility of sleeping in separate bedrooms in the event of a pandemic: yes *n *(%)	31 (100)

### Design

Semi-structured focus groups lasting between one to one and a half hours were conducted by a single group facilitator (the first author). In total one interview and eight focus groups were conducted; each group contained between two and six participants. Group discussions with two people do not constitute an ideal focus group design; only two groups were this small. However, when more than two participants were not able to meet at a convenient time, or when any participant failed to attend an arranged meeting, data collection proceeded with the number of participants present to avoid losing potentially valuable data. Separate focus groups were conducted with participants who had children (two groups) and participants who did not have children (six groups) as it was reasoned that parents might have different responses toward infection control measures given their particular responsibilities and circumstances. Six of the focus groups were mixed gender, with one group of men and one group of women. The focus groups were fairly homogenous with respect to age.

### Procedure

Approval for the study was granted by the University of Southampton Ethics Committee (study ID: 491) and Research Governance Office (reference: 5755). Data collection took place between May 2008 and July 2008. Written informed consent was obtained from all participants. A focus group schedule was used to guide the discussion, and a pilot interview (not included in the analysis) was undertaken to identify and rectify any problems with the schedule. The schedule first stimulated talk about perceptions of cold and influenza transmission and prevention. Participants were invited to discuss their thoughts about how colds and 'flu are caught and spread between people. Stimulus materials were then used to prompt discussion of handwashing, social distancing and cough hygiene as measures of reducing the spread of infections within households in the context of non-pandemic colds and influenza. The recommended infection control measures (see Table 2) were developed by the second author, in conjunction with a team of medical experts (see Acknowledgements).

**Table 2:** Recommended infection control measures

Handwashing:	
When ***someone in the house ***is infected:	When ***you ***are infected:
Wash your hands with soap or gel for **15 seconds **at least **6 times per day**	Wash your hands with soap or gel for **15 seconds **at least **once an hour**
Hands must be washed **before **you eat or drink anything	Hands must be washed **before **touching anything shared with other members of the household (e.g. door handles, TV remote controls etc.)
Hands must be washed **after **any contact with the infectious person or things they have touched	Hands must be washed **after **blowing your nose or covering your mouth when you cough

Social distancing:	

When ***someone in the house ***is infected:	When ***you ***are infected:
Keep at least **3 feet away **from the infected person	Keep at least **3 feet away **from other people
	Try not to spend too long in the same room as them

Cough hygiene:	

When ***you ***are infected:	
**Cover **your nose or mouth with a tissue every time you cough or sneeze	
**Throw the tissue away **at once in a disposable container (e.g. plastic bag)	

Participants were invited to discuss situations where they would or would not implement the measures, whether they thought the measures might be effective, and their feelings about the measures. The infection control measures were considered both in the event of someone else in the house becoming infected or if the participants themselves became infected. This was followed by a discussion of current knowledge and feelings about pandemic influenza. A definition of pandemic flu adapted from the UK Department of Health website [35] was provided to participants as a stimulus card (see Additional file 1). The group ended with a second discussion of the same infection control measures in the context of pandemic influenza. Neutral prompts were used to redirect or stimulate discussion where necessary.

### Data analysis

The focus groups and interview were audio recorded and transcribed verbatim. Inductive thematic analysis was then used to identify recurring patterns within the data [36], incorporating some techniques from grounded theory [37]. The analysis was undertaken from a broadly realist epistemology, assuming that what participants say largely reflects their genuine attitudes and experiences [38]. That said, the analysis does acknowledge that there may be social influences on participants' discussions, including group processes and perceived demands to give socially desirable responses. Data analysis was iterative and began after completion of the first six focus groups. Study enrolment ceased when no new themes emerged from the data i.e. when saturation had been achieved [39].

The analytic process consisted of four phases, following guidelines for conducting thematic analysis [40] and establishing validity in qualitative research [41]. Phase one involved familiarisation with the data by reading and re-reading all transcripts. Phase two involved first level open and 'in-vivo' coding (a technique taken from grounded theory), i.e. working line-by-line, each element of the text was given a code that summarised the meaning of that text segment, where possible using the participants' own words. In phase three these lower level codes were organised into potential themes. Constant comparison (another technique from grounded theory) was used to identify data that did not match the evolving coding scheme, which was then modified to fit the data better. Constant comparison was also used for 'deviant case analysis', i.e. to ensure that data that did not fit the dominant patterns was not ignored [41]. Discussions between the two authors shaped the development of the final coding structure. Phase four involved interpretation and exploration of the data, using the coding framework to identify the key influences on participants' likely adherence to infection control measures.

A paper trail was maintained throughout all phases of analysis documenting progression from the raw data to the findings reported here. Participants are identified by focus group (F) or interview (I) number, gender and age. Where quotations involve dialogue between two or more participants within a focus group, the participant (P) number is also given. The frequency with which each code occurred was noted, but is only reported qualitatively below (e.g. stating that a view was expressed by 'most', 'some' or 'a few' participants). Exact frequencies are not reported as how often a code was spontaneously expressed in our study cannot be taken as a reliable measure of how prevalent a view is in the population.

## Results

The final coding scheme consisted of 29 themes. These themes were grouped into three categories: knowledge and perceptions of infection transmission; attitudes towards infection control measures; and factors relating to the implementation of infection control measures (see Table 3). Themes related to knowledge and perceptions of infection transmission are only summarised briefly here. The analysis primarily focuses on the final two categories in order to provide a detailed review of potential influences on likely adherence to infection control measures. Although the views expressed by parents and non-parents did not appear to differ, the sample size does not permit firm conclusions to be drawn. Consequently, no explicit comparisons will be made between parents and non-parents within the results.

**Table 3:** Key themes

Knowledge and perceptions of infection transmission:	
Being near to someone who is infected	Catching an infection that is in the air
Touching an infected person or contaminated object	Not covering mouth or using a tissue when coughing or sneezing
Uncertainty about pandemic influenza	

Attitudes toward infection control behaviour:	

Positive attitude	Measures are ineffective
Responsibility for the wellbeing of yourself and others	Measures are inappropriate
Suggestion of alternative measures or compensatory behaviour	Selfishness
Negative attitude	Fear of becoming ill

Factors relating to implementation of infection control behaviour	

Normal routine	Practicality
Close or intimate relationships	Severity of the illness
Memory	Education

### Knowledge and perceptions of infection transmission

Participants were quite knowledgeable about the transmission routes of colds and influenza, including hand or other bodily contact with an infected person or contaminated object and airborne transmission by coughing and sneezing [42]. Knowledge levels did not appear to differ by age, gender or whether participants had children. However, there was much uncertainty about pandemic influenza. The limited knowledge participants did have centred on its serious and widespread nature. A large number of participants expressed distrust of some kind, which ranged from beliefs that pandemic influenza will not happen to a scepticism regarding the motives of the media and/or government:

"It's just scare mongering really it's like the end of the world was gonna be the year 2000 it just didn't happen it's probably never gonna happen so it's not a worry to me at all" (F9 male, age 20).

"Erm at this point in time I think that fear is kind of pumped out there and I get really annoyed when the media does that and I think they're just trying to make people get stuck in one place and not move around" (F2 female, age 24).

### Attitudes toward infection control measures

Positive attitudes were more often expressed toward handwashing and cough hygiene behaviours than to social distancing. Several participants agreed with the premise of the recommendations, believing the behaviours to be effective prevention measures. A few participants appreciated that such measures would provide a sense of control:

"I think it might give us a, give people anyway a sense of control over something we cannot control" (F4 female, age 26).

The favourable attitudes of all participants were markedly strengthened given the prospect of pandemic influenza. Older adults in particular stated they would go further than the suggested behaviours in such a scenario:

"You'd probably, you'd probably do more than that" (F5 male, age 49).

"Definitely, I'd be scrubbing me house down as well" (F8 female, age 49).

Responsibility for the protection of oneself or another appeared to be a strong motivation. This was especially evident given the prospect of pandemic influenza. Responsibility for the protection of others was presented as a stronger motivation than the protection of one's own health, especially amongst younger participants. Accordingly, motivation to protect one's own health was to be able to adequately care for others who became ill:

"Well yeah, obviously if you've picked up a disease and you're fighting it and nearly dying you're not gonna want to pass it on to your little sister or your younger brother or your mum or anyone are you?" (F6 male, age 23).

"Be more aware of other people and how they might get infected by you instead of relying on other people to protect themselves **from **you" (F9 male, age 18).

"It's really important to stay safe as you won't be able to care for them if you get ill" (F2 female, age 24).

Although participants were particularly motivated to protect the health of their family and loved ones, they also expressed a wider sense of responsibility to protect the health of any 'other' in society at risk of infection.

However, negative attitudes were also expressed in response to all of the recommended infection control behaviours. The recommendations were deemed difficult, hard or awkward to undertake, with a few participants suggesting their implementation would be impossible. Similarly, a large number of participants dismissed the behaviours as bothersome, inconvenient and not worth the effort:

"I don't think that anybody washes their hands more than what they already do. You only wash your hands at normal intervals that I think you would normally, like if you're eating, after you've been to the toilet, etcetera" (F8 male, age 52).

"I guess it's just a bit like, a bit of a hassle as well, just like thinking 'Oh, an hour's passed. I need to wash my hands"' (I3 female, age 19).

A few participants expressed concern about the stigma which may be associated with frequent handwashing:

"People might think you're a bit nuts if you did it outside the home – you'd get some funny looks wouldn't you?" (F5 male, age 49).

Over half of the participants questioned the effectiveness of the recommended infection control measures, believing that transmission of infection, particularly pandemic outbreaks, cannot be controlled and one must just "accept fate" (F10, female, age 24). This was a view primarily expressed by younger participants:

"I'd say um in terms of a pandemic it makes it sound like it's unstoppable anyway so if you're gonna get it you're gonna get it despite whatever you can do to try and help it it won't stop the pandemic and that's why it's called a pandemic" (F9 male, age 20).

Nearly all of the participants stated that some or all of the measures were unnecessary, too extreme or ineffective. Indeed, a common belief was that pandemic influenza would not originate in the UK, thus allowing scientists time to develop a vaccine:

"I'm not sure though 'cause if this is, if they don't even know what this flu is and they haven't found any vaccines or anything for it how do they know that antibacterial on your hands is gonna work? And just like washing your hands how do they know it's gonna stop, if you touch something oh it's alright I'll wash my hands how do they know it's gonna protect you?" (F7 male, age 23).

"P2: You've gotta think, I think we're a quite clean country compared to other places like I said it will be a less

P4: Developed

P2: Developed country I think that develops it first

P1: And that spreads it quicker

P4: And that will educate us

P1: We can get a vaccine off of them, sounds nasty but it's true, it's how the world works" (F6 P2: male, age 24, P4: male, age 23, P1: male, age 23).

Many participants believed that if somebody did contract a serious infection they would be treated in hospital, making infection control behaviours in the home redundant. Being too clean or too pedantic about hygiene was also considered detrimental to health. Indeed, a few participants stated that you should actually be worried if you did not catch a cold and that the immune system is strengthened by catching and fighting infections:

"P1: Yeah but I don't think you can be too um pedantic about it I mean if you're too clean I mean then you're

P3: Everything will jump on you

P1: Yeah yeah" (F5 P1: female, age 61, P3: male, age 49).

"P1: Oh yeah if you don't get a cold at least once a year there's something wrong with you" (F6 male, age 23)

Colds and seasonal influenza were considered 'normal' illness which did not pose a personal or serious threat to healthy individuals; only vulnerable individuals such as the elderly or very young need be concerned. Participants stated that infection control behaviours are only necessary for more serious, infectious diseases.

Selfish attitudes were prevalent in the context of non-pandemic influenza, suggesting that it was the responsibility of others to implement the behaviours.

"No 'cause I'd be expecting people looking after me to wash their hands 'cause I've already got it so what do I wanna keep washing me hands for (laughs)" (F8 female, age 44).

These selfish attitudes took two forms; either it is the responsibility of the ill person to protect others from infection, or it is the responsibility of the healthy person to protect themselves from becoming ill. In some instances these two contradictory attitudes were expressed by the same participant! A few participants stated that they did not feel motivated to undertake the recommended behaviours because they were concerned that other people would not be implementing them:

"But then I would think if I was to do this, the next, the next person isn't, why should I blow out the stops" (F6 male, age 24).

Participants frequently attempted to compensate for their negative attitudes by suggesting 'easier' behaviours such as the use of antibacterial gel, gloves or face masks. Alternatively, some participants said that they would devote more effort to the performance of one behaviour, particularly cleaning or handwashing, in order to compensate for the non-performance of other behaviours such as social distancing or not using tissues.

### Factors relating to the implementation of infection control measures

Participants suggested that behaviours already performed were more likely to be implemented. Commonly performed behaviours included: washing hands six times per day, particularly prior to eating; washing more regularly when ill; increasing one's distance from ill persons; and increasing one's distance when ill. A few participants considered cough hygiene measures "proper behaviour" (F4 female, age 26) which should be undertaken regardless of whether one is ill or not.

Social isolation of infected persons was not seen as an acceptable measure, especially if the infected person was a child. The need or desire to care for ill persons was seen as a major barrier to the implementation of social distancing measures. Indeed, some considered that it would be selfish to "flee" from an infected member of their family purely for self-protection:

"But don't you have some kind of duty, or at least I think I do to look after that person" (F9 male, age 24).

"What if it's a baby you've got to look after you can't do it can you? (F8 female, age 46).

In the context of a close or intimate relationship there was a lack of concern about being near to an infected loved one, and a fear of offending or insulting the infected person who would be in need of comfort when ill:

"P4: Well I wouldn't feel like just because you had the flu or a cold that I would have to

P1: Stay away

P4: Yeah have a different bedroom

P1: No

P4: I mean we would probably still share the same bed" (F5 P4: female, age 47, P1: female, age 61).

"I wouldn't want them to feel like isolated, and like they you know they couldn't come out and like socialise with us" (I3 female, age 19).

The impeding nature of a close relationship seemed strengthened at the prospect of an outbreak of pandemic influenza. Participants stated they would be reluctant to keep away from loved ones if either they or their loved one were dying of pandemic influenza:

"Oh man if you were dying I wouldn't, I'd like be at your side" (F10 female, age 24).

The need for memory joggers was advocated with many participants stating that even if they did wish to implement the measures they would most likely forget. This included, for example, handwashing timers to ensure hands were washed for an adequate length of time and adverts, posters or campaigns to remind people of the types of behaviours they should be undertaking. Indeed, many of the participants were not aware of the behaviours recommended to prevent the spread of colds and/or influenza between persons prior to participation in the study:

"No one's ever told you when, not even your doctor's told you when you get a cold you should wash your hands **a lot **more than you usually do" (F6 male, age 23).

Initiatives to improve understanding either at school, through doctors or in the media were considered valuable methods to facilitate implementation of the recommended behaviours.

Practical difficulties such as access to the required facilities represented one of the most commonly cited barriers to implementation. The use and disposal of tissues for every sneeze or cough was seen as a challenge given that coughs and sneezes are most often a surprise with disposal facilities not always nearby:

"P2: I still think your coughs, coughs and sneezes catch me by surprise

P1: Yeah you can't, you can't

P2: You're like that, or it's in your pocket or as you say you're in the living room by the time you've got to the toilet or wherever the tissue is you've done your load" (F6 P2: male, age 24, P1: male, age 23).

Other physical barriers to the implementation of the recommended behaviours included a lack of time to implement frequent handwashing and bodily effects such as sore noses from frequent tissue use or sore hands from frequent washing.

Despite all participants stating that it was possible for each member of their household to sleep in separate bedrooms, a lack of adequate space to maintain social distance was cited as a further practical barrier. This apparent contradiction appeared to be based on the assumption that the infected person would continue to be mobile. For example, several participants stated that maintaining social distance would not be feasible given the necessary, daily activities of the household e.g. eating, cleaning, moving between rooms etc. This assumed mobility may result from the perceived unacceptability of restricting an individual's movement within the household. As one participant states, a household represents "shared" space to which all members are entitled access:

"so yeh it's not that practical, especially if they're just wandering around the house like you have to share living space" (I3, female, age 19)

"I think it will still be quite practically hard if like the person that was infected chose just to walk around the house or flat like practically it's really hard to keep three feet away from them, maybe wait till they've walked out the corridor and just things like that" (F4, female, age 19)

It seems that, at least in the context of non-pandemic colds and influenza, socially isolating members of a household contradicts the ethos of what a household should be; a shared, socially connected and 'free' household.

The impact of illness, for example fatigue or energy loss, was viewed as a significant barrier, particularly in the context of pandemic influenza:

"P1: Yeah but would you be able to [wash regularly and decontaminate surfaces]?

P2: That's what I'm saying I still don't know

P4: Yeah somebody else would have to do it

P2: You'd be too ill. (F8 P1: female, age 49, P2: female, age 44, P4: female, age 46).

Maintaining implementation of all measures was considered highly unlikely in the event of persistent or prolonged illness. That said, all the participants concurred that if it was something very serious, such as pandemic influenza, they would be more determined to implement all of the infection control behaviours:

"I think you know when it's life or death situations you're gonna do whatever is recommended" (F4 female, age 19).

"If your life's at risk I think everyone would do it" (F6 male, age 23).

## Discussion

In general, participants were knowledgeable about the transmission routes of colds and influenza, supporting the previous work of Vingilis et al. [20]. Participants were less knowledgeable with regard to pandemic influenza, its origins and the threat it poses. These findings are in line with Janssen et al. [32], who reported low concern for pandemic influenza amongst the general public in the United States. In the context of non-pandemic influenza, infection control behaviours were met with unfavourable attitudes. Specifically, participants felt that the behaviours may do more harm than good and/or were unnecessary. However, participants expressed a much greater willingness to carry out hygiene behaviours in the context of pandemic influenza. Thus, participants reported that they were only likely to implement the recommended behaviours in the event of a serious health threat, reinforcing previous research suggesting the importance of perceived personal risk [43]. That said, pandemic health threats with symptoms perceived as mild e.g. 'swine 'flu', may not result in widespread and consistent implementation of infection control behaviours. Moreover, habit is an important influence on routine behaviour [44] – including hygiene behaviour [31] – and despite their best intentions people may find it difficult to implement new hygiene measures during a pandemic if they have not previously made these a habit.

A common motivation for implementing the behaviours was to protect the health of others. This is consistent with the views of the older Chinese people during the SARS epidemic [30], and suggests that the motivation to protect family members may not be a culture-specific phenomenon. That said, the role of social responsibility in infection control is a complex issue. For example, some participants also expressed selfish attitudes, passing responsibility for the implementation of infection control behaviours to other members of the household. Although such attitudes were more often expressed in the context of non-pandemic influenza, survey studies conducted during the SARS epidemic [13] suggest that selfish attitudes may also be influential in pandemic contexts. More research is required to understand the balance between the motivation to protect oneself, family and others and how these motivations will interact to influence behaviour in pandemic contexts.

Several factors were cited as likely to influence the ability to implement infection control behaviours. Practical difficulties such as access to facilities and skin irritation were similar to those cited by health care workers [22,23]. Participants stated they would be unable to undertake social distancing measures in the event of a pandemic due to the need to care for and remain close to other household members. This supports the findings of previous qualitative research conducted during the SARS epidemic [28]. Despite demonstrating adequate knowledge regarding infection transmission, several participants stated they would benefit from more comprehensive education and/or reminders regarding infection control behaviours.

Several of the themes identified within this study fit within the conceptual model proposed by Curtis, Danquah, and Aunger [31] to explain the handwashing practices of caregivers in developing countries. The implementation of infection control behaviours appears to depend on a number of environmental (e.g. time, energy, availability of facilities, social norms), motivational (e.g. social responsibility, social relationships, selfishness) and habitual (e.g. education, use of memory aids) factors.

Any conclusions drawn from this study must be considered tentative only due to a number of considerations. Although a fairly large and varied sample of participants was recruited, the sample size does not permit conclusions regarding the effect of sociodemographic factors such as age, gender, occupational background, relationship status or whether or not one has children. The effect of social influences on responses seemed evident in a minority of the discussions. For example, participants showed a tendency toward conforming to the responses of their friends, partner or spouse. It is also possible that different responses may have been expressed in individual interviews. There were some indications of 'groupthink' [45], particularly evident within male dominated discussions, where a strong consensus was reached within the group without consideration of alternative attitudes and opinions. This precludes conclusions based on gender as it is unknown whether these males would express the same attitudes to infection control behaviours outside of this group situation. Most importantly, given the hypothetical nature of the present study, it is quite possible that in the event of a pandemic individuals may behave entirely differently than anticipated, for better or for worse.

Further research is needed in order to confirm and/or develop the conclusions drawn. For example, it would be useful to verify and quantify the extent to which reservations about the acceptability and practicality of social distancing may pose a motivational barrier to this preventive behaviour. Attention should also be paid to identifying the role of sociodemographic factors and how they may differentially affect willingness to implement infection control behaviours. Future research should also seek to validate the extent to which the factors mentioned by participants really influence behaviour, and to empirically test whether attitudes and behaviour can be changed by intervention.

## Conclusion

Despite the limitations outlined, our findings may be of value when designing infection control interventions. Of the behaviours likely to influence infection transmission, handwashing was regarded by our participants as more feasible than cough and sneeze hygiene and more acceptable than social distancing, and so may be a useful target for health promotion. Consequently, encouraging handwashing will form the key focus of our web-based intervention, since attempting to promote behaviours that people are unable or unwilling to engage is naturally more likely to provoke scepticism and low 'self-efficacy' – a self-fulfilling anticipation of failure [46]. Interventions may also need to address a number of beliefs identified in this study as potential barriers to carrying out infection control behaviours. For example, it may be necessary to persuade members of the community that pandemic influenza could pose a real threat to those living in developed western countries, that infection control measures can be effective, and that it is important to protect oneself from infection in order to be able to care for family members. In addition to these motivational arguments, practical measures may be needed to support implementation, such as education, reminders and provision of hand gel.

## Competing interests

The authors declare that they have no competing interests.

## Authors' contributions

LM contributed to the design, carried out the data collection and analysis, and drafted the manuscript. LY conceptualized and supervised the project, discussed emerging codes, checked the final coding, and edited the manuscript. Both authors read and approved the final manuscript.

## Pre-publication history

The pre-publication history for this paper can be accessed here:



## Supplementary Material

Additional file 1 **Description of pandemic influenza**. A definition of pandemic influenza provided to the participants as a stimulus card during the focus groups and interview. Click here for file
