# Supplementary material for: What infection control measures will people carry out to reduce transmission of pandemic influenza? A focus group study
Source: BMC Public Health. 2009 Jul 23;9:258. doi: 10.1186/1471-2458-9-258 (PMC2720966; doi:10.1186/1471-2458-9-258)
Supplement: Additional file 1 — Description of pandemic influenza. A definition of pandemic influenza provided to the participants as a stimulus card during the focus groups and interview. [file 1471-2458-9-258-S1.pdf]

Description of pandemic influenza

What is pandemic influenza?

Colds and flu are so common that we usually do not try to avoid them, since it is rare to become seriously ill. But experts agree that an outbreak of a new strain of flu is likely to occur soon, which could cause a worldwide pandemic.

You need to know how to reduce the risk of you and your family catching pandemic flu because everyone will be at risk; no-one will be immune:

- Pandemic flu is much worse than normal flu; it may kill healthy people,
- There may not be time to develop a vaccine to protect people,
- Anti-viral medication may be less effective than usual,
- If you catch the flu you may give it to your family,
- If you catch the flu from family members you may be unable to care for them while you are ill.
